# Mitochondrial dysfunction in age-related sarcopenia: mechanistic insights, diagnostic advances, and therapeutic prospects

**DOI:** 10.3389/fcell.2025.1590524

**Published:** 2025-10-03

**Authors:** Yingtao Huang, Chenchen Wang, Haijian Cui, Guangjiang Sun, Xiaonan Qi, Xiaosheng Yao

**Affiliations:** ^1^ The First Clinical College, Liaoning University of Traditional Chinese Medicine, Shenyang, China; ^2^ Department of Orthopedics, The First Affiliated Hospital of Liaoning University of Traditional Chinese Medicine, Shenyang, China; ^3^ The Second Clinical College, Liaoning University of Traditional Chinese Medicine, Shenyang, China

**Keywords:** sarcopenia, muscle atrophy, aging, mitochondrial dysfunction, chronic inflammation, therapeutic strategies

## Abstract

Sarcopenia is a progressive age-related decline in skeletal muscle mass, strength, and function, representing a significant health burden in older adults. Diagnostic criteria have been established that integrate measures of muscle mass, strength, and physical performance [e.g., European Working Group on Sarcopenia in Older People 2010 (EWGSOP1) and 2019 (EWGSOP2) criteria]. Mechanistically, sarcopenia is driven by hormonal changes, chronic inflammation, cellular senescence, and, importantly, mitochondrial dysfunction. Age-related declines in sex hormones and activation of myostatin impair muscle regeneration and metabolism, while chronic low-grade inflammation disrupts protein synthesis and accelerates proteolysis via the ubiquitin–proteasome system (UPS) and autophagy–lysosome pathway (ALP). The accumulation of senescent cells and their secretory phenotype further exacerbates muscle degeneration and functional decline. Mitochondrial dysfunction plays a central role, characterized by impaired biogenesis, excessive reactive oxygen species (ROS) production, compromised autophagy/mitophagy, and accumulation of mitochondrial DNA (mtDNA) mutations. These defects collectively disrupt muscle energy homeostasis, promoting atrophy. The AMPK/SIRT1/PGC-1α and mTORC1 signaling pathways, along with PINK1/Parkin-mediated and receptor-dependent mitophagy, are essential for regulating mitochondrial biogenesis, protein synthesis, and mitochondrial quality control. Current and emerging therapeutic approaches include resistance and endurance exercise, nutritional and pharmacological agents targeting mitochondrial health, and hormonal modulation. Innovative treatments such as senolytics, exerkines, and gene therapies show promise but require further validation. Future advances in mechanistic understanding, diagnostics, and therapeutic strategies offer hope for mitigating sarcopenia and improving the quality of life in aging populations.

## 1 Introduction

Sarcopenia, derived from the Greek words sarx (“flesh”) and penia (“loss”), refers to the progressive and generalized loss of skeletal muscle mass, strength, and function associated with aging (Rosenberg, 2011; Cruz-Jentoft and Sayer, 2019; Bahat and Ozkok, 2024). Originally coined by Irwin Rosenberg in the 1980s to describe age-related muscle wasting (Rosenberg, 1989), the term now encompasses a broader spectrum of muscle degeneration, including declines in muscle quality and performance. Sarcopenia has been recognized as a major public health concern due to its strong associations with increased risks of a range of adverse health outcomes, such as falls, physical disability, loss of independence, hospitalization, and mortality among older adults (Cruz-Jentoft et al., 2019). Epidemiological studies indicate that its prevalence varies widely, ranging from 9.9% to 40.4% in community-dwelling elderly adults, largely depending on the diagnostic criteria and assessment methods employed (Mayhew et al., 2019). Diagnosis typically involves the assessment of muscle mass (e.g., by DXA or bioimpedance), muscle strength (commonly grip strength), and physical performance (e.g., gait speed or chair stand test), as standardized by international working groups, such as the European Working Group on Sarcopenia in Older People (EWGSOP) 2010 (EWGSOP1) (Cruz-Jentoft et al., 2010) and the Asian Working Group for Sarcopenia (AWGS) 2014 (AWGS 2014) (Chen et al., 2014). The growing clinical and public health significance of sarcopenia is driving research to develop novel prevention and treatment strategies, which necessitates a deeper understanding of its underlying cellular pathogenesis to identify specific therapeutic targets.

Aging is a multifactorial process that underlies the progressive decline in skeletal muscle mass and function, central to the development of sarcopenia. Mitochondrial dysfunction and impaired mitophagy have emerged as critical drivers of this deterioration (Ryu et al., 2020; Triolo et al., 2022; Liao et al., 2023). As key regulators of energy production, redox balance, and metabolic signaling, mitochondria are vital for maintaining muscle fiber integrity and plasticity (Marzetti et al., 2013). However, aging leads to structural and functional mitochondrial impairments, including diminished biogenesis, accumulation of damaged mitochondria, and reduced respiratory capacity, resulting in compromised energy metabolism, elevated ROS generation, and mitochondrial DNA (mtDNA) damage—all of which accelerate muscle atrophy and weakness.

In parallel, aging disrupts protein degradation systems such as the ubiquitin–proteasome system (UPS) and the autophagy–lysosome pathway (ALP), leading to the buildup of dysfunctional proteins and organelles (Seo et al., 2016). Mitophagy, the selective autophagic clearance of damaged mitochondria, plays a central role in preserving mitochondrial quality. Its decline promotes oxidative stress, chronic inflammation, and muscle degeneration, contributing to sarcopenia progression (Triolo et al., 2022; Liao et al., 2023). In addition to energy metabolism, mitochondria govern calcium signaling and apoptotic pathways, further underscoring their importance in muscle cell survival (Youle and Narendra, 2011). The accumulation of defective mitochondria from insufficient mitophagy has been identified as a hallmark of aging skeletal muscle (Nichenko et al., 2021), although mechanistic links between mitochondrial dysregulation and sarcopenia remain incompletely understood.

**FIGURE 1:**
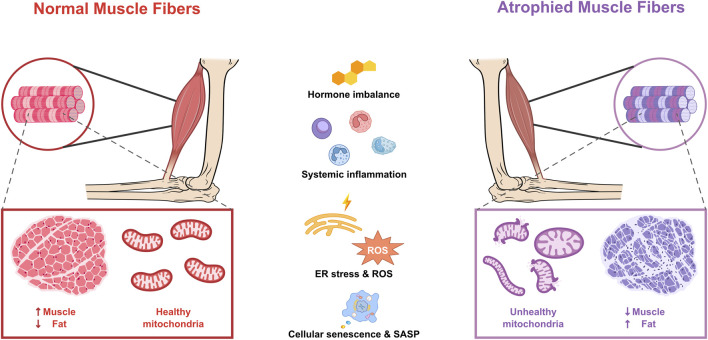
Mitochondrial dysfunction and age-related factors in atrophied muscle fibers. Adenosine triphosphate (ATP); endoplasmic reticulum (ER); reactive oxygen species (ROS); senescence-associated secretory phenotype (SASP).

This review illustrates the relevant mechanisms proposed to underlie the relationship between aging, muscle mitochondrial dysfunction, and sarcopenia (Figure 1). It begins with a brief overview of the evidence linking mitochondrial dysfunction to human muscle aging. The review then discusses prominent mitochondrial pathways implicated in the pathogenesis of sarcopenia. Furthermore, it highlights that aging and mitochondrial dysfunction may serve as promising therapeutic targets, offering hope for mitigating sarcopenia and improving the quality of life in aging populations.

## 2 Diagnostic criteria and prevalence of sarcopenia

The current understanding of sarcopenia involves not only muscle mass but also muscle strength and physical performance. Accurate diagnostic frameworks and prevalence data are therefore critical for early identification of individuals at risk and the implementation of effective intervention strategies.

### 2.1 Diagnostic methods and criteria

Sarcopenia has a profound effect on the health, daily functioning, and overall quality of life of older adults. Detecting the condition early and providing timely support can reduce its negative impact and support more active, independent aging. Diagnosis typically relies on evaluating muscle strength, quantity, and physical performance, with several international expert groups providing standardized guidelines (Table 1).

**TABLE 1:** Summary of the diagnostic criteria for sarcopenia.

Criteria	Strength measure	Muscle mass measure	Physical performance	Diagnostic classification
EWGSOP1 (2010) criteria (Cruz-Jentoft et al., 2010)	Handgrip <30 kg (men)/< 20 kg (women)	ASM/height^2^< 7.26 kg/m^2^(men)/< 5.50 kg/m^2^(women)	4 m gait speed ≤0.8 m/s	• Presarcopenia: low musclemass only • Sarcopenia: low muscle mass+ (low strength or lowperformance) • Severe sarcopenia: low mass+ low strength + lowperformance
EWGSOP2 (2019) criteria (Cruz-Jentoft et al., 2019)	Handgrip <27 kg (men)/< 16 kg (women) or 5-CST time ≥15 s	ASM/height^2^< 7.0 (men)/< 6.0 kg/m^2^(women) or ASM <20 kg (men)/15 kg (women)	Any of • Gait speed ≤0.8 m/s • SPPB ≤8 • TUG ≥20 s • 400 m walk ≥6 min	• Probable sarcopenia: lowstrength only • Confirmed sarcopenia: lowstrength + low muscle mass • Severe sarcopenia: lowstrength + low mass + lowperformance
IWGS (2011) criteria (Fielding et al., 2011)	–	ASM/height^2^≤ 7.23 kg/m^2^(men)/≤ 5.67 kg/m^2^(women)	4 m gait speed ≤1.0 m/s	• Sarcopenia: low muscle mass+ low physical performance
FNIH (2014) criteria (Studenski et al., 2014)	Handgrip <26 kg (men)/< 16 kg (women)	ALM/BMI <0.789 (men)/< 0.512 (women)	Gait speed ≤0.8 m/s	• Sarcopenia: low strength +low muscle mass
AWGS 2014 criteria (Chen et al., 2014)	Handgrip <26 kg (men)/< 18 kg (women)	DXA-measured ASM/height^2^< 7.0 kg/m^2^(men)/< 5.4 kg/m^2^(women) or BIA-measured ASM/height^2^< 7.0 kg/m^2^(men)/< 5.7 kg/m^2^(women)	Gait speed <0.8 m/s	• Sarcopenia: low muscle mass+ (low strength or lowperformance)
AWGS 2019 update (Chen et al., 2020)	• Screening: calf circumference<34 cm (men)/< 33 cm(women) orSARC-F ≥ 4 or SARC-CalF≥11 • Possible: handgrip <28(men)/< 18 kg (women) or6MWT <1.0 m/s or SPPB ≤9or 5-CST time ≥12 s	Same as AWGS 2014	Same as AWGS 2014	• Possible sarcopenia • Confirmed sarcopenia: lowmass + low strength • Severe sarcopenia: low mass+ low strength + lowperformance

Abbreviations: European Working Group on Sarcopenia in Older People (EWGSOP); International Working Group on Sarcopenia (IWGS); Foundation for the National Institutes of Health Sarcopenia Project (FNIH); Asian Working Group for Sarcopenia (AWGS); appendicular skeletal muscle mass (ASM); appendicular lean mass (ALM); body mass index (BMI); dual-energy X-ray absorptiometry (DXA); bioelectrical impedance analysis (BIA); Short Physical Performance Battery (SPPB); Timed Up and Go Test (TUG); five-time chair stand test (5-CST); 6-min walk test (6MWT); Strength, Assistance with walking, Rise from a chair, Climb stairs, and Falls (SARC-F); SARC-F, combined with calf circumference (SARC-CalF).

The European Working Group on Sarcopenia in Older People first published diagnostic criteria in 2010 (EWGSOP1) (Cruz-Jentoft et al., 2010) and revised them in 2019 (EWGSOP2) (Cruz-Jentoft et al., 2019). The EWGSOP2 framework redefines sarcopenia as a progressive muscle disease that can begin earlier in life and is driven by factors beyond chronological aging. It elevates low muscle strength as the primary diagnostic parameter, with muscle quantity and physical performance serving as supportive measures. Clear cutoff points are provided to streamline both diagnosis and treatment.

The International Working Group on Sarcopenia (IWGS) focused on the decline in lean body mass (LBM) and physical performance (Fielding et al., 2011). In patients who are bedridden, unable to stand up from a chair without help, or whose gait speed over a 4 m course is under 1.0 m/s, clinicians should suspect sarcopenia. Individuals meeting any of these criteria should undergo body composition assessment via dual-energy X-ray absorptiometry (DXA), applying the current diagnostic thresholds for sarcopenia. A diagnosis requires both poor physical function (gait speed <1 m/s) and low fat-free mass indexed to height^2^, specifically an appendicular lean mass (ALM)/height^2^ of ≤7.23 kg/m^2^ for men and ≤5.67 kg/m^2^ for women.

The Foundation for the National Institutes of Health (FNIH) Sarcopenia Project established clinically relevant criteria for diagnosing sarcopenia by analyzing data from 26,625 community-dwelling older adults across nine studies (Studenski et al., 2014). Recommended diagnostic thresholds are grip strength <26 kg for men and <16 kg for women, and ALM adjusted for body mass index (BMI) (ALM/BMI) < 0.789 for men and <0.512 for women. These evidence-based criteria, validated through broad participant diversity, aim to aid clinical identification and trials targeting older populations at risk of functional limitations.

The AWGS first published its diagnostic criteria in 2014 (AWGS 2014) to reflect the ethnic and regional variations in body composition and functional capacity among Asian populations (Chen et al., 2014). Sarcopenia is defined as age-related loss of skeletal muscle mass accompanied by low muscle strength and/or poor physical performance. Cut-off values for DXA-measured ASM index (ASM/height^2^) are <7.0 kg/m^2^ for men and <5.4 kg/m^2^ for women, whereas for BIA-measured ASM index (ASM/height^2^), the thresholds are <7.0 kg/m^2^ for men and <5.7 kg/m^2^ for women. Handgrip strength thresholds are <26 kg for men and <18 kg for women. Physical performance is classified as poor when gait speed is <0.8 m/s, or alternatively, when the Short Physical Performance Battery (SPPB) score is ≤9 or the Timed Up and Go Test (TUG) time is ≥12 s. In response to growing clinical and research interest, the AWGS 2019 update (Chen et al., 2020) retained its established muscle-mass cut-offs but revised the diagnostic algorithm and strength/performance thresholds. The new criteria specify handgrip strength <28 kg for men and <18 kg for women, gait speed <1.0 m/s in the 6-min walk test, a Short Physical Performance Battery score ≤9, or a five-time chair stand test (5-CST) time ≥12 s. This definition also introduced “possible sarcopenia” (low strength or low performance alone) for early primary-care intervention. It also introduced distinct algorithms for community and hospital settings, both of which initiate screening with one of the following measures—calf circumference (<34 cm for men and <33 cm for women), Strength, Assistance with walking, Rise from a chair, Climb stairs, and Falls (SARC-F) questionnaire (≥4), or SARC-F combined with calf circumference (SARC-CalF) questionnaire (≥11)—to enable earlier risk identification.

Regional variations in mainstream sarcopenia diagnostic criteria largely reflect differences in body habitus, muscle composition, lifestyle factors, healthcare infrastructure, and the underlying epidemiological data of each population. Compared with Asian criteria (AWGS 2014 and AWGS 2019), European and North American guidelines (EWGSOP1, EWGSOP2, and FNIH) use more stringent muscle-strength cutoffs, reflecting both resource availability and larger reference cohorts. Lifestyle and functional dependencies, such as walking speed, chair-rise performance, and community support structures, also vary by region and influence the weighting of physical-performance tests in each algorithm. Each working group derives its thresholds and prognostic models from locally recruited prospective studies, so diagnostic criteria and predictive accuracy inevitably differ. This reflects a precision-medicine approach in which sarcopenia identification is customized to the demographic profiles and real-world conditions of specific populations.

### 2.2 Prevalence and risk factors

Sarcopenia is a common condition among older adults, with prevalence rates varying depending on the studied population and diagnostic criteria used. Among individuals aged 60 years and older, prevalence estimates derived from studies employing all six established sarcopenia classification systems (EWGSOP1, EWGSOP2, AWGS 2014, IWGS, FNIH, and based on muscle mass only) range from 10% to 27% (Petermann-Rocha et al., 2022). According to the widely accepted definition of sarcopenia established by the EWGSOP (EWGSOP1), the prevalence of sarcopenia is estimated to range from 5% to 13% among individuals aged 60–70 years and from 11% to 50% among those older than 80 years (Haase et al., 2022). According to Xia et al. (2024), the prevalence of sarcopenia among hospitalized older adults (aged ≥65 years) was 46.4%. Compared with their non-sarcopenic counterparts, these patients tended to be older, had a lower BMI, and presented with more comorbidities.

Alterations in circulating hormones are significantly associated with changes in women’s skeletal muscle mass, strength, and body fat across a continuum of ages (Critchlow et al., 2025). Several researchers have examined the prevalence of sarcopenia by gender. In a cross-sectional study conducted in a rural area of Eastern China, Yang et al. (2022) found that the prevalence of sarcopenia was higher in women than in men. This finding aligns with the results of a study by Tay et al. (2015), which reported higher prevalence rates of sarcopenia among elderly women compared with men. In contrast, a study by Kirchengast and Huber (2009) observed that among healthy elderly individuals, men over 80 years had a higher prevalence of sarcopenia (50%) compared with women of the same age group (43.8%). These discrepancies suggest the need for further research to clarify gender-specific prevalence rates of sarcopenia across different populations and age groups.

Regional differences contribute to variations in the prevalence of sarcopenia. A systematic review (Petermann-Rocha et al., 2022) of regional prevalence estimates revealed considerable variability both across continents and according to the diagnostic criteria used. The highest sarcopenia prevalence was reported in Oceania when applying the EWGSOP1 definition (40%), whereas South American studies relying on muscle-mass thresholds alone found rates of approximately 35%. By contrast, the lowest estimates emerged in Europe under the more stringent EWGSOP2 criteria (1%) and in Oceania when using the FNIH algorithm (5%). African estimates remain confined to FNIH-based studies, which suggest a prevalence of about 13% (Petermann-Rocha et al., 2022).

Studies also indicate that sarcopenia is more prevalent in non-Asian than in Asian populations. For example, women in non-Asian countries have a prevalence of 20% when measured using BIA, compared with 11% in Asian countries. These disparities may be attributed to lifestyle differences, such as healthier dietary habits and higher levels of physical activity compared with Western populations, both of which serve as protective factors against sarcopenia (Shafiee et al., 2017).

In a Japanese study, former elite athletes such as Olympic participants had about 50% lower odds of sarcopenia in their seventies compared with community controls. This effect was attributed to sustained intense training that helps maintain limb muscle mass and strength. Paradoxically, they also suffered more frequent declines in physical function and greater musculoskeletal pain, particularly among contact-sport athletes, pointing to the necessity of early promotion of intense exercise and the development of targeted safety and rehabilitation interventions to preserve function and minimize pain in old age (Tanaka et al., 2021).

## 3 Impact of aging on sarcopenia

Age-driven hormonal declines and increased myostatin levels contribute to anabolic resistance, impairing mammalian/mechanistic target of rapamycin (mTOR)-mediated protein synthesis and shifting the balance toward muscle degradation. Hyperactivity in the UPS and ALP, compounded by chronic low-grade inflammation, accelerates proteolysis and disrupts regenerative signaling. Additionally, the accumulation of senescent cells and their pro-inflammatory secretions compromises satellite cell function and neuromuscular junction integrity, thereby advancing sarcopenia.

### 3.1 Hormonal changes

Aging is typically accompanied by significant hormonal changes in both men and women. In particular, the decline in sex hormones directly affects muscle mass and strength.

Testosterone binds to androgen receptors in both myonuclei and satellite cells and also shapes muscle metabolism by modulating the release and activity of various cytokines and signaling molecules (Shin et al., 2018; Shigehara et al., 2022). As men age and circulating testosterone levels decline, muscle mass and strength deteriorate due to diminished receptor-mediated stimulation of muscle fibers and satellite cell proliferation, as well as alterations in cytokine profiles and metabolic pathways. These physiological changes manifest as slower gait, impaired balance, increased frailty, and a heightened risk of falls (Shigehara et al., 2022).

In post-menopausal women, estrogen deficiency during and after menopause severely impairs the survival, self-renewal, and differentiation of skeletal muscle satellite cells, leading to their loss and compromised muscle regeneration (Wosiak et al., 2021). Menopause-associated low serum estradiol levels can greatly contribute to declines in muscle mass, strength, and physical performance, thereby increasing the risk of developing sarcopenia in women. Although factors such as body composition, nutrient intake, and metabolic indicators also play roles in muscle health, estrogen deficiency remains a pivotal underlying mechanism (Rathnayake et al., 2021). Critchlow et al. (2025) investigated both cross-sectional and longitudinal associations between circulating sex hormones and skeletal muscle mass and function in women aged 24–89 years. They found that circulating estradiol (E2) and free estradiol index (FEI) were positively correlated with relative appendicular lean mass (ALM) and thigh muscle percentage. In contrast, these hormones showed a negative correlation with total body fat.

Myostatin is a growth factor that inhibits muscle growth by limiting muscle cell proliferation and differentiation. Research has indicated that age-related hormonal changes, including reductions in estrogen and testosterone, may activate myostatin, exacerbating muscle loss (Zheng et al., 2024). The interaction between hormonal changes and myostatin could be a crucial factor in the development and progression of sarcopenia.

### 3.2 Imbalance between protein synthesis and degradation

The development of sarcopenia is closely linked to an imbalance between muscle protein synthesis and muscle protein degradation. As individuals age, this imbalance tends to shift in favor of protein degradation, leading to the progressive loss of muscle mass. Sarcopenia is partly driven by anabolic resistance, a condition in which aging skeletal muscle becomes progressively less responsive to anabolic stimuli, such as protein intake and resistance exercise. This diminished responsiveness impairs muscle protein synthesis, ultimately contributing to muscle loss (Breen and Phillips, 2011; Aragon et al., 2023).

Skeletal muscle protein balance is determined by the difference between muscle protein synthesis and breakdown. Age-related sarcopenia in older adults (>60 years) results primarily from decreased resting myofibrillar protein synthesis, approximately 20%–30% lower than in younger individuals, coupled with an increase of up to 50% in markers indicative of muscle protein breakdown, leading to a more negative net protein balance compared with younger adults (Breen and Phillips, 2011). If the decrease in resting fractional synthesis rates and the increase in myofibrillar breakdown were to occur simultaneously as indicated, the resultant muscle loss would exceed the typically observed annual rate of 0.5%–1.5% seen in individuals aged 50–80 years (Hughes et al., 2001). However, later studies failed to consistently demonstrate markedly lower muscle protein synthesis or substantially higher muscle protein breakdown in healthy older subjects (Volpi et al., 1998; Volpi et al., 2001; Cuthbertson et al., 2005), leading to the consensus that basal skeletal muscle net protein balance is generally maintained with aging (Volpi et al., 2001). It should be recognized that current methods for assessing muscle protein turnover might lack sufficient sensitivity to detect subtle yet important changes in elderly adults. Moreover, discrepancies between earlier and later studies may arise from challenges in accurately differentiating robust seniors from their frail counterparts. Frailty in older individuals, often associated with increased systemic inflammation, multiple comorbidities, or lower levels of physical activity, can disrupt the metabolism of skeletal muscle proteins (Toth et al., 2005; Degens, 2010).

At the molecular level, signaling proteins associated with the mTOR pathway have consistently been shown to respond to amino acids, particularly leucine. The diminished sensitivity of aged muscle to anabolic stimuli may be attributed to impaired phosphorylation of mTOR-mediated signaling proteins (Breen and Phillips, 2011; Aragon et al., 2023). This impaired sensitivity results in a less pronounced increase in muscle protein synthesis following protein ingestion (Wall et al., 2015; Wu et al., 2023). Lower physical activity, commonly observed in older adults, further exacerbates declines in muscle protein synthesis. Inactivity leads to a loss of muscle mass and strength, promoting a cycle of increasing frailty and decreased physical activity (Wall et al., 2015).

### 3.3 Activation of proteolytic systems

During skeletal muscle atrophy, key proteolysis systems and molecules, including the UPS, ALP, calpain system, and caspase-3 (CASP3), play significant roles in protein degradation. The UPS targets misfolded or defective proteins for degradation through a process involving ubiquitination, which is mediated by E3 ligases such as muscle atrophy F-box protein (MAFbx, also known as atrogin 1 or F-box only protein 32 [FBXO32]) and muscle RING finger protein 1 (MuRF1), leading to muscle mass loss. In conditions like aging, injury, and chronic disease, the UPS becomes overactive, disrupting protein homeostasis and causing protein aggregation and redox imbalance. The ALP, involved in chaperone-mediated autophagy, microautophagy, and macroautophagy, also regulates muscle atrophy by degrading cellular components via autophagosomes and lysosomal hydrolases. Forkhead Box Protein O3 (FoxO3) and BCL2/adenovirus E1B 19 kDa-interacting protein 3 (BNIP3) play key roles in regulating autophagy during atrophy. Calpains and CASP3 work upstream of the UPS to degrade myofibrillar proteins, and their activity is interconnected, with oxidative stress and inflammation exacerbating muscle atrophy. The combined action of these proteolytic systems accelerates muscle degradation, particularly in conditions of oxidative stress or inflammation (Chen et al., 2023).

### 3.4 Chronic inflammation

Chronic inflammation, also known as inflammaging, is a hallmark of aging and a major driver of sarcopenia. In older adults, persistent low-grade inflammation disrupts muscle metabolism by shifting the balance between protein synthesis and degradation. Proinflammatory cytokines such as tumor necrosis factor (TNF)-α and interleukin (IL) 6 (IL6) inhibit muscle protein synthesis by interfering with the mTOR signaling pathway (Wu et al., 2023). Moreover, TNF-α activates the UPS to degrade myogenic factors such as myogenic differentiation 1 (MyoD) (Ogawa et al., 2016) and exacerbates muscle atrophy by modulating the protein kinase B (Akt)/mTOR/FoxO axis and inducing atrophy-related ubiquitin ligases (Hunter and Bailey, 2019). To further elucidate TNF-α’s role in muscle regeneration, Alvarez et al. (2024) treated proliferating C2C12 myoblasts with TNF-α and performed RNA sequencing, identifying 958 differentially expressed genes enriched in TNF-α, chemokine, and IL17 signaling pathways. Transcripts for IL6, the C-C motif chemokine ligand 7 (CCL7), and matrix metalloproteinases (MMP) 9, 10, and 13 (MMP9, MMP10, and MMP13) were markedly upregulated, along with the extracellular matrix regulator versican and the myogenic inhibitor myostatin. At the protein level, Myf5 increased while MyoD decreased, indicating that TNF-α drives a transcriptional program that promotes myoblast proliferation and extracellular matrix remodeling while delaying differentiation. These processes facilitate regeneration after injury. However, when chronically activated, they may accelerate sarcopenic decline.

Chronic inflammation significantly contributes to muscle degeneration observed in age-related sarcopenia. It primarily alters the composition of muscle fibers by boosting the proportion of type I fibers while decreasing the percentage of fast-twitch type II fibers, especially type IIB. Type II fibers are more susceptible to protein degradation induced by inflammatory cytokines like TNF-α, leading to muscle atrophy and loss of strength and endurance. TNF-α specifically impairs muscle regeneration by decreasing the expression of crucial myogenic regulatory factors, such as MyoD, and promoting MyoD degradation via the ubiquitin-proteasome pathway (Sciorati et al., 2020).

TNF-α and interferon (IFN)-γ exert convergent inhibitory effects on MyoD in myogenic cells. Low-dose TNF-α activates c-Jun N-terminal kinase 1 (JNK1) signaling to induce both LIF secretion and myostatin transcription, which together suppress MyoD protein levels and block myoblast differentiation (Alvarez et al., 2024). Likewise, IFN-γ-driven activation of Janus kinase (JAK) 1/2 (JAK1/2) pathways as a key mechanism impairing muscle satellite cell function, promoting senescence, and causing myofiber atrophy in inclusion body myositis (Hou et al., 2025). Elevated levels of TNF-α bind to TNF receptors 1 and 2 (TNFR1 and TNFR2), leading to chronic activation of NF-κB and MAPK signaling pathways and disruption of the phosphoinositide 3-kinase (PI3K)/Akt pathway in muscle cells. By promoting pyroptosis and inflammation, this signaling cascade accelerates muscle protein degradation and inhibits protein synthesis, ultimately driving age-related muscle loss (Cheng et al., 2025).

It should be noted that MyoD expression is nearly undetectable in resting adult skeletal muscle but is rapidly upregulated following injury to initiate muscle regeneration. However, under conditions promoting muscle atrophy, MAFbx targets MyoD for proteasomal degradation, further exacerbating muscle loss. Preventing this degradation, either by disrupting the interaction between MAFbx and MyoD or by expressing a ubiquitination-resistant mutant form of MyoD (MyoDK133R), has been shown to preserve MyoD levels and mitigate muscle atrophy *in vivo* (Lagirand-Cantaloube et al., 2009).

Additionally, inflammaging contributes not only to muscle degradation but also to impaired muscle function. The chronic inflammatory environment characteristic of aging reduces the efficiency of muscle contraction, diminishes muscle force generation, and adversely affects overall muscle performance. Furthermore, aberrant activation of fibro-adipogenic progenitors (FAPs) in muscle tissue drives fibrosis and fatty infiltration, thereby intensifying muscle atrophy and functional decline (Chen et al., 2022). Recent research has increasingly emphasized the critical role of muscular fatty infiltration in the age-related decline of skeletal muscle function, with growing evidence suggesting a strong connection between this factor and physical inactivity (Brioche et al., 2016).

### 3.5 Cellular senescence

Cellular senescence is a biological process in which cells enter a state of irreversible growth arrest (Grimes and Chandra, 2009). Although senescent cells remain metabolically active, they lose the ability to replicate. Increasing evidence suggests that the buildup of these cells in skeletal muscle plays a major role in age-related declines in muscle mass and function. Senescent cells exhibit permanent arrest of the cell cycle, extensive chromatin remodeling, and a pro-inflammatory secretome known as the senescence-associated secretory phenotype (SASP). These cells arise in both mononuclear progenitor populations and fully differentiated myofibers (Englund et al., 2021). The factors released through the SASP create a persistently inflamed environment that disrupts tissue homeostasis and regeneration, ultimately leading to muscle atrophy and weakness.

**FIGURE 2:**
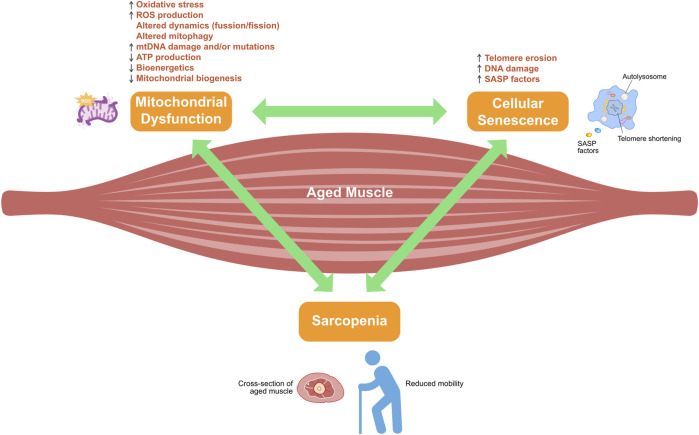
Crosstalk between mitochondrial dysfunction and cellular senescence in sarcopenia development. Adenosine triphosphate (ATP); mitochondrial DNA (mtDNA); reactive oxygen species (ROS); senescence-associated secretory phenotype (SASP).

Compared with the reference group, middle-aged adults with sarcopenic obesity display elevated adiposity, reduced muscle mass, and markedly shorter telomeres (Goddard et al., 2022). In skeletal muscle cells, excessive mitochondrial ROS contribute to the accumulation of single-strand breaks in telomeric DNA, thereby accelerating telomere erosion and triggering cellular senescence (von Zglinicki et al., 2000). By counteracting mitochondrial ROS generation, telomere shortening slows, myocyte lifespan is prolonged, and muscle homeostasis is restored (Passos et al., 2007), ultimately delaying the onset of sarcopenia (Figure 2).

Motor neuron fibers innervate myofibers at neuromuscular junctions to maintain muscle mass and health, and muscle satellite cell activation near regenerating neuromuscular junctions is critical for proper myofiber reinnervation and for limiting denervation-induced atrophy and fibrosis (Sousa-Victor et al., 2022). Recent single-cell and bulk transcriptomic analyses have identified a distinct subpopulation of fibro-adipogenic progenitors in aged murine muscle that express high levels of p16^INK4a^ and other senescence markers, accompanied by DNA damage and chromatin changes (Zhang X. et al., 2022). Similarly, a subset of old myofibers exhibits a p21^CIP1^-driven senescence program, suggesting that both supporting stromal cells and the muscle fibers themselves can contribute to the senescent burden. Importantly, pharmacological clearance of senescent cells in aged mice reverses molecular hallmarks of senescence, improves muscle morphology, and enhances strength, underscoring the therapeutic promise of senolytic interventions (Zhang X. et al., 2022).

The role of senescence in muscle adaptation and repair appears to be nuanced, with transient senescent-like states potentially facilitating regeneration after injury, particularly in younger individuals (Dungan et al., 2023). However, persistent accumulation of senescent cells in aged muscle impairs the adaptive response to exercise and blunts repair processes. Emerging data even suggest that multinucleated muscle fibers themselves can enter a senescent-like state, further exacerbating functional decline (Dungan et al., 2023).

## 4 Role of mitochondrial dysfunction in the development of sarcopenia

Mitochondrial dysfunction is a significant contributor to the pathophysiology of sarcopenia. Mitochondria are the powerhouses of the cell, responsible for producing the energy required for various cellular processes through oxidative phosphorylation. In muscle cells, this energy production is critical for maintaining muscle function and mass. With aging, several changes occur in mitochondrial function that contribute to sarcopenia.

### 4.1 Increased ROS production

In age-related sarcopenia, the increased production of reactive oxygen species (ROS) plays a central role in driving cumulative oxidative damage in skeletal muscle cells, and this process is further exacerbated by uncontrolled inflammation. ROS are by-products of mitochondrial metabolism (Chen et al., 2023).

Superoxide dismutase (SOD) is universally recognized as the first line of defense against the toxicity of superoxide (O_2_^•−^), catalyzing the dismutation of two superoxide molecules into hydrogen peroxide (H_2_O_2_) and molecular oxygen (O_2_), thereby limiting O_2_^•−^ availability (Rosa et al., 2021). Sullivan-Gunn and Lewandowski (2013) found that age-related sarcopenia in mice is associated with increased oxidative stress in skeletal muscle, likely driven by elevated NADPH oxidase (NOX) activity and reduced antioxidant protection. At 18 months, when sarcopenia first appears, NOX expression and superoxide levels rise alongside increased Sod1 activity, indicating a compensatory antioxidant response. However, this is accompanied by higher hydrogen peroxide (H_2_O_2_) levels and decreased catalase and Gpx activity, suggesting weakened antioxidant defense. By 24 months, NOX and superoxide remain high, but Sod1 activity declines without further muscle loss. These findings suggest that NOX-mediated ROS production, especially H_2_O_2_, and impaired antioxidant enzymes play key roles in the development of sarcopenia.

Moreover, skeletal muscle atrophy is characterized by a state of uncontrolled inflammation and oxidative stress that exacerbates proteolytic metabolism (Ma et al., 2018; Yadav et al., 2021; Zhang et al., 2023). Inflammatory cytokines, including IFN-γ, IL6, transforming growth factor (TGF)-β, and TNF-α, are released during muscle injury and repair (Londhe and Guttridge, 2015; Ji et al., 2022), and their presence, together with redox signaling dysregulation from membrane-localized nicotinamide adenine dinucleotide phosphate (NADPH) oxidases (Jackson, 2015; Pascual-Fernández et al., 2020), creates a feed-forward loop in which chronic inflammation further activates NADPH oxidase and other inducible enzymes, promoting additional ROS production (Mukund and Subramaniam, 2020). Activation of toll-like receptor 2 (TLR2) has also been shown to induce oxidative stress and inflammation, while its inhibition can attenuate skeletal muscle atrophy (Kim et al., 2015). Furthermore, compounds like salidroside and *Tinospora cordifolia* extract protect against skeletal muscle atrophy by inhibiting oxidative stress and inflammation, suppressing proteolytic and autophagic pathways, and promoting muscle differentiation and growth in denervation and nutrient deprivation models (Huang et al., 2019; Wu et al., 2019; Sharma et al., 2020).

Concurrently, endoplasmic reticulum (ER) stress induced by the accumulation of unfolded or misfolded proteins activates the unfolded protein response (UPR) (Bohnert et al., 2018). C/EBP Homologous Protein (CHOP) is upregulated by UPR, and it promotes apoptosis by altering the transcription of genes involved in cell death and oxidative stress while relieving protein kinase R (PKR)-like endoplasmic reticulum kinase (PERK)-mediated translational inhibition to enhance the production of pro-apoptotic proteins. The key transcriptional targets include B-cell lymphoma 2 homology domain 3 (BH3)-only proteins B-cell lymphoma 2-like 11 (BIM), p53 upregulated modulator of apoptosis (PUMA), and NADPH oxidase activator (NOXA), along with ER oxidoreductase (ERO)-1α and tribbles pseudokinase 3 (TRIB3) (Logue et al., 2013). These proteins promote apoptosis by increasing pro-apoptotic factors and suppressing anti-apoptotic proteins like BCL2. The increased expression of BH3-only proteins and repression of anti-apoptotic proteins like BCL2 lead to apoptosis by facilitating BCL2-associated X protein (BAX)/BCL2 antagonist/killer 1 (BAK) homo-oligomerization, mitochondrial outer membrane permeabilization, cytochrome c release, and apoptosome formation (Deldicque, 2013).

Sustained ER stress disrupts anabolic signaling pathways, notably the mTOR complex 1 (mTORC1) complex, which is crucial for muscle protein synthesis and regeneration. This disruption contributes to a condition known as anabolic resistance, where the muscle’s ability to synthesize proteins in response to stimuli is diminished, particularly in older individuals (Deldicque, 2013). Furthermore, ER stress activates redox-sensitive pathways such as nuclear factor kappa-light-chain-enhancer of activated B cells (NF-κB), which plays a pivotal role in inflammation. NF-κB activation leads to the expression of pro-inflammatory cytokines like TNF-α and IL6, which can promote muscle protein breakdown through the ubiquitin-proteasome pathway. This inflammatory response is exacerbated by oxidative stress, creating a vicious cycle that accelerates muscle degradation (Agrawal et al., 2023). Cyclooxygenase (COX) 2 (COX2) is an inducible isoform of cyclooxygenase, which is minimally expressed under normal physiological conditions. However, its expression significantly increases in response to inflammatory stimuli, playing a key role in the inflammatory response and potentially causing tissue damage. COX2 promotes prostaglandin synthesis, and this further contributes to inflammation (Zhang L. et al., 2022). Thus, the interaction between oxidative stress, inflammation, and ER stress creates a vicious cycle that accelerates neuromuscular degeneration, muscle fiber loss, and the progression of sarcopenia.

### 4.2 Impaired mitochondrial biogenesis

Mitochondrial homeostasis is maintained by the intricate balance between mitochondrial biogenesis, which replenishes healthy mitochondria, and mitophagy, which selectively removes damaged organelles (Chen et al., 2023). Disrupting this balance can result in the accumulation of dysfunctional mitochondria, thereby compromising cellular energy metabolism and upsetting the equilibrium between protein synthesis and degradation, especially in skeletal muscle (Lei et al., 2024). Additionally, mitochondrial dysfunction contributes to skeletal muscle atrophy by increasing ROS production, reducing mitochondrial biogenesis, disrupting mitochondrial dynamics, and impairing mitophagy (Lei et al., 2024). Consequently, mitochondrial impairment not only exacerbates energy deficits but also activates multiple signaling pathways that enhance proteolysis and inhibit protein synthesis, thus intensifying muscle atrophy (Kubat et al., 2023).

Peroxisome proliferator-activated receptor gamma coactivator (PGC)-1α (also abbreviated as PPARGC1A) is one of the master regulators of mitochondrial biogenesis and quality control in skeletal muscle. It activates the transcription of mtDNA by initiating a cascade involving nuclear respiratory factors (NRFs) (NRF-1, NRF-2) and estrogen-related receptors (ERR) (ERR-α), ultimately promoting the expression of mitochondrial transcription factor A (TFAM), which regulates mtDNA replication and transcription (Popov, 2020). Additionally, sirtuin 1 (SIRT1) activates PGC-1α through deacetylation, and this activated PGC-1α then interacts with TFAM to facilitate its transport into the mitochondria, where it forms a complex with the D-loop region of mtDNA (Abu Shelbayeh et al., 2023). TFAM can bind and coat mtDNA, protecting it from ROS and degradation while increasing mitochondrial function (Theilen et al., 2017). The critical role of PGC-1α is evidenced by studies in which skeletal muscle–specific deletion of Pgc-1α in mice resulted in diminished mitochondrial biogenesis markers and concomitant reductions in whole-body performance. The interaction between PGC-1α and ERR-α stimulates mitochondrial fusion (Shally and McDonagh, 2020). Reduced induction of PGC-1α and its weakened cooperation with NRFs and ERR-α critically underlie the age-related decline in mitochondrial biogenesis and dynamics.

SOD2 (manganese superoxide dismutase) is a critical antioxidant enzyme that protects skeletal muscles from oxidative stress during aging. In mouse models, skeletal muscle-specific PGC-1β deficiency downregulates Sod2 expression, leading to elevated oxidative stress, mitochondrial dysfunction, and reduced endurance exercise capacity (Gali Ramamoorthy et al., 2015). Sod2 functions by scavenging O_2_^•−^ produced by the mitochondrial respiratory chain, thus preventing oxidative damage to muscle fibers. The reduced Sod2 levels in PGC-1β-deficient muscles exacerbate muscle fatigue and oxidative damage. PGC-1β overexpression, on the other hand, enhances Sod2 expression, alleviating oxidative stress and maintaining muscle function (Gali Ramamoorthy et al., 2015). Therefore, Sod2 is essential in mitigating age-related skeletal muscle decline by controlling oxidative stress and supporting mitochondrial integrity.

### 4.3 Decline in muscle autophagy

Autophagy is a fundamental cellular process responsible for degrading and recycling damaged organelles and misfolded proteins, thereby maintaining cellular homeostasis and responding to various stresses (Khandia et al., 2019). Sarcopenia is also significantly influenced by the impairment of autophagic processes during aging. Although initially thought to promote muscle atrophy, recent research indicates that autophagy is vital to maintain muscle mass, with its function notably decreasing in aged muscles. Enhanced autophagic activity has been shown to counteract muscle aging by facilitating the selective removal of dysfunctional organelles and misfolded proteins, thereby protecting against age-associated muscle deterioration (Jiao and Demontis, 2017). Conversely, reduced autophagic activity exacerbates mouse muscle loss and strength decline, as demonstrated by studies where deletion of key autophagy-related genes, such as autophagy related (Atg) 5 (Atg5) and Atg7, led to severe muscle atrophy, abnormal mitochondrial accumulation, and disorganization of muscle fibers (Masiero et al., 2009). Thus, preserving autophagy flux is critical to mitigate the effects of aging on skeletal muscle.

Moreover, autophagy confers systemic anti-aging benefits that extend beyond skeletal muscle by regulating myokines—muscle-derived signaling proteins that modulate whole-body homeostasis and inflammatory responses. This systemic communication suggests that maintaining optimal autophagy could be a promising therapeutic target not only for sarcopenia but also for extending healthspan more broadly (Jiao and Demontis, 2017). Furthermore, autophagy protects against sarcopenia by enhancing the regenerative capacity of satellite cells, reducing oxidative stress, and suppressing inflammation, which are key pathological features of age-related muscle dysfunction (Xie et al., 2023).

### 4.4 Regulatory role of mitophagy

Mitophagy, the selective autophagic degradation of damaged mitochondria, affects mitochondrial integrity and function. Mitochondrial dysfunction, particularly disruptions in mitochondrial dynamics and mitophagy, can contribute to muscle atrophy. Although the mechanisms are still being explored, it is clear that maintaining proper mitochondrial quality control through mitophagy is essential for preventing muscle degeneration (Lei et al., 2024).

BNIP3 and NIP3-like protein X (NIX, also known as BNIP3-like [BNIP3L]) are proteins crucial for mitochondrial autophagy (mitophagy) in skeletal muscle. They function as compensatory mitophagy mediators when the primary PINK1 pathway is deficient or impaired. In oxidative muscle tissues, such as the soleus, loss or dysfunction of PINK1 triggers an upregulation of BNIP3/NIX expression, thereby maintaining mitochondrial homeostasis and ensuring adequate mitophagy levels (Singh et al., 2024).

While mitophagy is generally protective, excessive or insufficient mitophagic activity can exacerbate muscle wasting. Immobilization-induced muscle atrophy leads to mitochondrial dysfunction, with decreased mitochondrial respiration and elevated lipid peroxidation. This is accompanied by increased mitophagy, suggesting a compensatory mechanism to prevent further myofiber damage. However, antioxidant treatment or inhibition of autophagy suppresses mitophagy, leading to greater muscle atrophy and mitochondrial dysfunction. In contrast, enhancing mitophagic flux through interventions like urolithin A has shown beneficial effects on muscle function, underscoring the protective role of mitophagy in preventing muscle degeneration during disuse. These findings suggest that maintaining mitophagic activity is essential for preserving muscle integrity and combating atrophy (Rahman et al., 2025).

In contrast to declining autophagy, mitophagy appears preserved or even elevated in aged mouse tissues (Jiménez-Loygorri et al., 2024; Rappe et al., 2024). Nevertheless, swollen and dysfunctional mitochondria accumulate, leading to the release of mtDNA that activates the cyclic GMP–AMP synthase (cGAS)/stimulator of interferon genes (STING) pathway. The Geriatric group displayed the greatest activation of cGAS/STING pathway components and downstream phospho-interferon regulatory factor 3 (Irf3) targets, along with a pronounced enrichment of interferon (IFN-α and IFN-γ) response and TNF-α signaling compared with all other groups (Jiménez-Loygorri et al., 2024). Pink1/Parkin-mediated mitophagy spikes in aged tissues but is insufficient to prevent organellar damage, and genetic ablation of Pink1 or Parkin worsens inflammation unless STING is also removed (Jiménez-Loygorri et al., 2024). Given the complexity of the regulatory mechanisms underlying mitophagy and its intricate interplay with other forms of autophagy, attaining a comprehensive understanding of mitophagy remains a significant challenge.

### 4.5 mtDNA deletions and duplications

Mitochondrial dysfunction in skeletal muscle, driven by the accumulation of large-scale mtDNA deletions and duplications, may lead to significant impairments in oxidative phosphorylation. This is evidenced by the emergence of COX-deficient (COX^–^) fibers and ragged red fibers, which indicate severe respiratory chain defects and a compensatory upregulation of mitochondrial biogenesis (Wanagat et al., 2001; Herbst et al., 2007). Despite the presence of up to 17% COX^–^fibers in some muscles, satellite cell-mediated regeneration appears to counterbalance the localized damage, preserving overall muscle mass and functional performance (Bua et al., 2002; Spinelli and Haigis, 2018).

However, the impact on satellite cells themselves is more detrimental. Muscle satellite cells are specialized adult stem cells in skeletal muscle that remain mostly inactive until they are triggered by injury to proliferate and differentiate, thereby repairing and regenerating muscle fibers. Freshly isolated muscle satellite cells expressing K320E-Twinkle demonstrate increased mitochondrial biogenesis with greater mitochondrial area and higher PGC-1α expression, whereas proliferating cells show reduced mitochondrial mass, lower mtDNA copy number, and diminished respiratory chain levels due to a selective process of mitochondrial turnover that removes damaged mtDNA. During muscle regeneration, the rapid expansion of the mitochondrial pool derived from mutated mtDNA leads to the accumulation of mtDNA alterations. This results in the formation of COX-deficient (COX^−^) fibers, muscle fiber atrophy, and impaired recovery of muscle mass, a pattern that closely mirrors the progression of human sarcopenia (Kimoloi et al., 2022).

### 4.6 Mitochondrial characteristics in aging slow- and fast-twitch fibers

In skeletal muscle, the transition from rest to contraction demands a dramatic upregulation of ATP synthesis, with energy requirements rising nearly 100-fold during active cross-bridge cycling. Readily available ATP stores fuel only ∼2 s of continuous contraction, while phosphocreatine buffering extends this by another ∼10 s. To sustain longer bouts of activity, muscles recruit both anaerobic glycolysis and, critically, mitochondrial oxidative phosphorylation. At submaximal intensities, oxidative metabolism predominates; however, as exercise intensity surpasses a certain threshold, anaerobic pathways increasingly contribute to ATP production to match the heightened energy flux (Marzetti et al., 2024).

Mitochondrial density, spatial distribution, and ultrastructure differ markedly between slow-twitch type I and fast-twitch type II fibers. Type I fibers possess dense, highly interconnected networks of subsarcolemmal and intermyofibrillar mitochondria, underpinning their superior fatigue resistance but limiting peak force generation due to smaller fiber and motor unit size. By contrast, type II fibers, which can be further subdivided into IIa (oxidative) and IIx (glycolytic), exhibit progressively lower mitochondrial content and larger cross-sectional areas, conferring high force production at the expense of rapid fatigability. With aging, these intrinsic architectural and metabolic distinctions become accentuated. Fast-twitch fibers undergo a more pronounced decline in mitochondrial volume, cristae integrity, and oxidative enzyme activity, rendering them particularly vulnerable to age-related atrophy, whereas slow-twitch fibers retain greater mitochondrial functionality and structural connectivity, partially preserving their endurance capacity despite senescent stressors (Marzetti et al., 2024). However, appropriate endurance or resistance exercise can stimulate mitochondrial biogenesis and quality control in type II fibers, partially restoring their size and metabolic function and attenuating their degenerative transformation (Long et al., 2022).

## 5 Pathways involved in the crosstalk between aging and mitophagy in sarcopenia development

The interaction between mitochondrial dysfunction and aging is pivotal in understanding the development and progression of sarcopenia. Aging contributes to skeletal muscle degeneration in part by impairing mitophagy, the selective degradation of damaged mitochondria (Triolo and Hood, 2021). Mitophagy is tightly regulated by several pathways. During aging, dysregulation of these pathways leads to a decrease in both autophagic and mitophagic activity. This dysregulation contributes to the accumulation of damaged mitochondria, thus promoting muscle loss and weakness (Urbina-Varela et al., 2020; Leduc-Gaudet et al., 2021) (Table 2).

**TABLE 2:** Key signaling pathways involved in the crosstalk between aging and mitophagy in sarcopenia development.

Pathway	Function and mechanism	Relation to sarcopenia development
AMPK/SIRT1/PGC-1α pathway	AMPK is activated under energy stress conditions (e.g., starvation, exercise) and regulates mitochondrial biogenesis, dynamics, and mitophagy through the PGC-1α signaling pathway. SIRT1, an NAD^+^-dependent deacetylase, modulates the oxidative stress response by deacetylating targets like FOXO3, which enhances antioxidant defenses and mitochondrial function	Aging impairs this pathway, contributing to sarcopenia by reducing mitochondrial function, autophagy, and cellular repair processes. This leads to the accumulation of cellular damage and mitochondrial dysfunction, resulting in muscle weakness and loss of mass
mTORC1 pathway	mTORC1 functions as a nutrient-sensitive suppressor of autophagy. Under nutrient-rich conditions, mTORC1 inhibits autophagy by phosphorylating ULK1, which reduces ULK1’s kinase activity and its ability to interact with cofactors (ATG13 and FIP200). This prevents the initiation of autophagy. During energy deprivation, AMPK inhibits mTORC1, causing it to dissociate from the ULK1 complex. This allows ULK1 to activate and trigger the autophagy process	mTORC1 signaling can promote protein synthesis and inhibit autophagy under basal conditions. mTORC1 inhibition caused by excessive AMPK activation can reduce protein synthesis and enhance autophagy, potentially contributing to muscle atrophy
Ubiquitin-dependent mitophagy pathway (PINK1/Parkin pathway)	Damaged mitochondria accumulate PINK1 on the outer membrane, recruiting PARKIN to ubiquitinate mitochondrial proteins and marking them for autophagic degradation	The PINK1/Parkin pathway becomes dysregulated during aging and chronic low-grade inflammation (inflammaging), thereby exacerbating mitochondrial dysfunction and contributing to muscle loss
Receptor-dependent mitophagy pathways mediated by BNIP3/NIX and FUNDC1	BNIP3, NIX, and FUNDC1 promote mitophagy through receptor-mediated mechanisms: BNIP3 and NIX induce mitochondrial fission by interacting with DRP1, regulate mitochondrial calcium homeostasis, and modulate the balance between apoptosis and necrosis; FUNDC1 initiates mitophagy primarily via post-translational modifications, especially phosphorylation	Increased BNIP3 and NIX expression during aging initially helps counteract mitochondrial damage. However, their overactivation can lead to excessive mitophagy and cell death, worsening the progression of sarcopenia. Changes in FUNDC1 activity, particularly during muscle unloading, may contribute to mitochondrial loss and subsequent muscle atrophy

### 5.1 AMPK/SIRT1/PGC-1α and mTORC1 signaling pathways

Adenosine 5′-monophosphate (AMP)-activated protein kinase [AMPK, also known as protein kinase AMP-activated alpha 1 catalytic subunit (PRKAA1)] is a central regulator of cellular energy homeostasis, activated under conditions of energy stress such as starvation or exercise (Dossou and Basu, 2019). AMPK and SIRT1 are cellular energy sensors that detect shifts in energy status. Activated AMPK and SIRT1 can directly promote PGC-1α activity through phosphorylation and deacetylation, respectively (Yang et al., 2024). PGC-1α subsequently drives the transcription of genes essential for oxidative metabolism and mitochondrial maintenance. This integrated network optimizes energy expenditure, enhances metabolic fitness, and preserves muscle function throughout the aging process (Ferri et al., 2020). The AMPK/SIRT1/PGC-1α pathway in skeletal muscle is thought to reduce mitochondrial dysfunction and mitigate sarcopenia (Tian et al., 2019).

Activation of PGC-1α enhances mitochondrial biogenesis, optimizes mitochondrial metabolism, and mitigates oxidative stress-induced injury in skeletal muscle. SIRT1, an NAD^+^-dependent deacetylase, modulates the oxidative stress response by sensing intracellular NAD^+^ levels and deacetylating targets such as FOXO3, thereby activating antioxidant defenses and preventing cell death (Li et al., 2024). Resveratrol increases NAD^+^ bioavailability to stimulate SIRT1, which in turn deacetylates LKB1, promoting its phosphorylation and subsequent AMPK activation at Thr^172^ (Cantó and Auwerx, 2012).

AMPK emerges as a master regulator of mitochondrial homeostasis, linking fission to mitophagy and, under prolonged energy stress, relaying signals to the nucleus to drive the biogenesis of new mitochondria and replace damaged ones (Toyama et al., 2016). AMPK is activated by low nutrients/stress and directly phosphorylates UNC-51-like kinase (ULK) 1 (ULK1, also known as ATG1)/ULK2 to initiate autophagy, including mitophagy. Loss of AMPK or ULK1 leads to defective mitophagy with the abnormal accumulation of p62. Moreover, an ULK1 mutant that can’t be phosphorylated by AMPK fails to maintain mitochondrial homeostasis and support cell survival during starvation (Egan et al., 2011).

AMPK also modulates autophagy by phosphorylating the transcription factor FOXO3, particularly at the Ser^588^ residue. This phosphorylation stabilizes and activates FOXO3 within the nucleus, thereby promoting transcription of essential autophagy and ubiquitin-proteasome pathway genes, including MAFbx, MuRF1, microtubule-associated protein 1 light chain 3 (LC3), ULK1, and ATG13. Although FOXO3 does not directly interact with ATG complexes, its role as a transcriptional activator is crucial for initiating autophagy. This transcriptional response facilitates the degradation and recycling of cellular components during metabolic stress, thereby protecting against muscle degeneration. Nevertheless, chronic or dysregulated activation may lead to excessive proteolysis and consequent muscle wasting (Sanchez et al., 2012).

**FIGURE 3:**
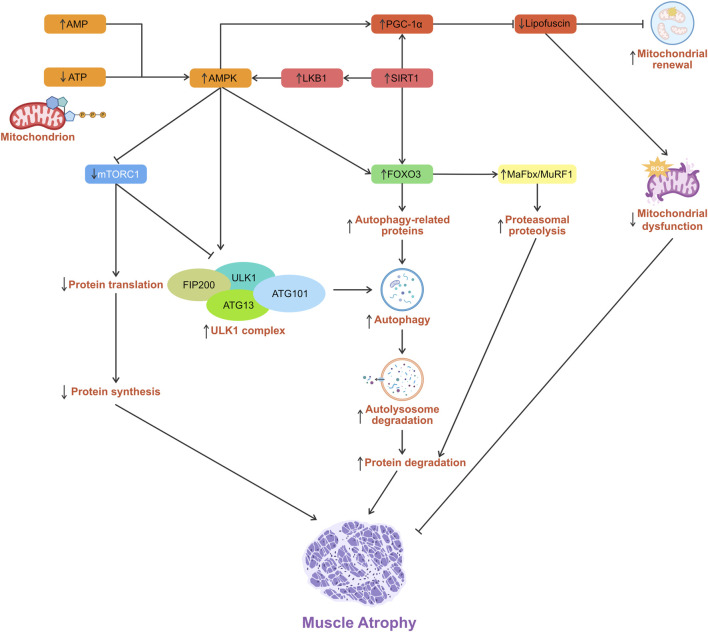
Schematic diagram showing the role of AMPK in the regulation of skeletal muscle atrophy.

Beyond transcriptional regulation through FOXO3, AMPK further regulates autophagy by inhibiting mTORC1 signaling, a key nutrient-sensitive suppressor of autophagy. In mammalian cells, the ULK1 complex, which consists of ULK1 (autophosphorylation), ATG13, FAK family kinase-interacting protein of 200 kDa (FIP200), and ATG101, plays a central role in initiating autophagy. Under nutrient-rich conditions, mTORC1-mediated repressive phosphorylation of ULK1 modulates its kinase activity and/or its interaction with the cofactors ATG13 and FIP200, thereby coordinating the autophagy response (Sanchez et al., 2012). During energy deprivation, AMPK activation leads to mTORC1 inhibition and its dissociation from the ULK1 complex (Dossou and Basu, 2019). The activated ULK1 complex then translocates to the ER isolation membrane, initiating autophagy (Puente et al., 2016; Zachari and Ganley, 2017). mTORC1 signaling can promote protein synthesis and inhibit autophagy under basal conditions. mTORC1 inhibition caused by excessive AMPK activation can reduce protein synthesis and enhance autophagy, potentially contributing to muscle atrophy (Sanchez et al., 2012) (Figure 3).

Age-related mitochondrial dysfunction drives a progressive loss of cellular bioenergetic capacity, most notably reflected by a decrease in maximal oxygen uptake. This decline results from both a decrease in mitochondrial number and impaired respiratory function, creating an energy shortfall that compromises muscle performance and resilience (Short et al., 2004).

### 5.2 Mitophagy pathways

Mitophagy pathways are classified into two main types: the ubiquitin-dependent pathway mediated by PINK1 and Parkin, and the receptor-mediated pathway involving BNIP3, NIX, and the mitochondrial protein FUN14 domain-containing 1 (FUNDC1) (Fritsch et al., 2020). Under conditions of mitochondrial damage, PINK1 accumulates on the outer mitochondrial membrane, recruiting and activating Parkin, an E3 ubiquitin ligase. This activation leads to the ubiquitination of mitochondrial proteins, marking them for degradation via autophagy. PINK1/Parkin pathway is vital for mitochondrial quality control and cellular homeostasis (Seabright and Lai, 2020; Liu et al., 2021). Inflammaging (chronic low-grade inflammation associated with aging) exacerbates mitochondrial dysfunction and contributes to sarcopenia (Xu and Wen, 2023).

Oral administration of the AMPK activator MK-8722 significantly activated AMPK in mouse gastrocnemius muscle, leading to increased mitolysosome numbers and enhanced mitophagy. This effect was shown to be dependent on the Pink1/Parkin pathway, as it was absent in Pink1^−/−^ mice. Notably, while Ampk activation reduced mitophagy in other tissues, it specifically promoted Pink1/Parkin-dependent mitophagy in skeletal muscle, highlighting that distinct mitophagy pathways dominate in different tissues and respond differently to Ampk activation (Longo et al., 2024).

Mitophagy can also be initiated by mitochondrial-localized receptors such as BNIP3, NIX, and FUNDC1, which stimulate interactions with LC3/GABA_A_ receptor-associated protein (GABARAP) (Stotland and Gottlieb, 2015; Wei et al., 2015; Yamaguchi et al., 2016). BNIP3 and NIX are pro-apoptotic proteins of the BH3-only subfamily of the BCL2 protein family. The two molecules are primarily localized to the outer mitochondrial membrane and play significant roles in regulating apoptosis and mitochondrial function (Chinnadurai et al., 2008). Under stress conditions, they can induce mitophagy by binding to the mitochondrial membrane, selectively clearing damaged or redundant mitochondria to maintain mitochondrial function and energy metabolism within cells (Wang et al., 2023).

Age-related accumulation of mtDNA damage is compounded by the impaired function of key mitophagy regulators such as BNIP3L, NIX, and FUNDC1, which fail to efficiently clear damaged mitochondria in aging muscle (Hepple, 2014; Lampert et al., 2019). The increased expression levels of BNIP3 and NIX, typically during aging, can promote mitophagy to counteract mitochondrial damage. Specifically, BNIP3 and NIX interact with the mitochondrial fission protein dynamin-related protein 1 (DRP1) to induce mitochondrial fission, thereby facilitating the occurrence of mitophagy (Lee et al., 2011). This process is particularly important in the development of sarcopenia, as mitochondrial dysfunction is a critical factor in this disease (Doblado et al., 2021). BNIP3 and NIX promote calcium transfer from the ER to mitochondria by displacing BCL-2 from the inositol 1,4,5-trisphosphate (IP3) receptor and voltage-dependent anion channels (VDACs) at the mitochondrial-associated membranes (MAMs). This mechanism leads to ER calcium depletion, mitochondrial calcium accumulation, and mitochondrial permeability transition (MPT), potentially contributing to cell death pathways such as necrosis or apoptosis (Field and Gordon, 2022). Additionally, the excessive activation of BNIP3 and NIX may lead to overactive mitophagy, causing cell death, which poses a potential risk for muscle cells in sarcopenia patients (Urbina-Varela et al., 2020).

FUNDC1-mediated mitophagy is primarily activated through post-translational modifications, including phosphorylation, rather than by changes in its protein expression. This mechanism remains incompletely characterized in muscle unloading (Liu et al., 2012; Wu et al., 2014; Chen et al., 2016; Lv et al., 2017). A mouse study (Leermakers et al., 2019) found reduced Fundc1 protein levels in the skeletal muscles of unloaded mice, despite unchanged mRNA expression, indicating that Fundc1 protein abundance is regulated post-translationally during the initial phases of unloading. Although Fundc1 depletion may contribute to mitochondrial loss, the direct causal relationship still requires further investigation. Skeletal muscle-expressed FUNDC1 serves as a pivotal regulator of fat utilization and exercise capacity. It is also involved in mitochondrial quality control and maintenance of metabolic homeostasis (Fu et al., 2018). Loss of FUNDC1 in muscle disrupts mitophagy and compromises mitochondrial energy production, resulting in mitochondrial defects and diminished athletic performance (Fu et al., 2018; Gan et al., 2018; Triolo and Hood, 2021).

## 6 Therapeutic strategies for treating sarcopenia

In recent years, numerous therapeutic approaches have been explored to counteract this debilitating condition. These interventions, spanning hormonal therapies to natural compounds, have each demonstrated varying degrees of promise.

### 6.1 Targeting aging and inflammation

#### 6.1.1 Exerkines

Exerkines, including myokines, are primarily secreted by contracting skeletal muscle fibers and exert autocrine, paracrine, and endocrine effects, a concept recently illuminated by studies on muscle contraction during and after exercise (Piccirillo, 2019). In the aging context, these exercise-induced factors enhance skeletal muscle energy supply, activate satellite cells that drive repair and regeneration, improve neuromuscular junction integrity, and support neural control of muscle function, collectively combating sarcopenia (Wang et al., 2025).

Among the most studied exerkines, IL6 exhibits dual actions. Chronically elevated IL6 levels contribute to muscle atrophy, while the transient rise triggered by exercise promotes myogenic differentiation and muscle regeneration (Fischer, 2006; Wu et al., 2019). Apelin released upon contraction improves muscle contractility and reduces oxidative stress in aged mice, positioning it as a therapeutic candidate against myofibrillar atrophy and weakness (Luo et al., 2021; McKenna et al., 2022). Decorin, expressed in muscle and other tissues, inhibits myostatin signaling to prevent proteolysis and fiber wasting (Kawaguchi et al., 2020), and IL15 enhances local energy metabolism in myofibers while protecting against high-fat diet-induced obesity, glucose intolerance, and insulin resistance (Nadeau and Aguer, 2019; Nadeau et al., 2019). Targeting the myostatin/activin A axis remains a potent strategy for muscle wasting as blockade of activin type II receptors yields robust hypertrophy and functional gains. Additionally, musclin has emerged as an exercise-induced myokine that stimulates mitochondrial biogenesis and endurance capacity (Subbotina et al., 2015).

#### 6.1.2 Compounds

Drug repurposing has gained attention as a cost-effective strategy for treating sarcopenia. Medications used for other conditions, such as type 2 diabetes, may possess pro-anabolic or anti-inflammatory properties that could benefit sarcopenia patients. For instance, metformin, a commonly prescribed medication for type 2 diabetes, can inhibit pro-inflammatory cytokine production (including IL1 and TNF-α) and block nuclear factor kappa-light-chain-enhancer of activated B cells (NF-κB) signaling (Kim and Choi, 2012; Gu et al., 2014), while also elevating circulating irisin levels (Li et al., 2015). Moreover, it suppresses skeletal muscle senescence by modulating mitochondrial complex I activity and the AMPK, mTOR, and NF-κB signaling pathways, thereby enhancing muscle mass, strength, and endurance while reducing pro-inflammatory cytokine levels in aged or sarcopenic mouse models (Fielder et al., 2022; Lyu et al., 2022). Other diabetes medications, such as glucagon-like peptide-1 (GLP-1) receptor agonists, dipeptidyl-peptidase 4 (DPP4) inhibitors, and sodium-glucose cotransporter 2 (SGLT2) inhibitors, may also be beneficial in treating sarcopenia (Hardee and Lynch, 2019; Witham et al., 2023; Bahat and Ozkok, 2024).

Natural compounds with anti-aging properties also hold potential for sarcopenia treatment. Ursolic acid (UA) has shown protective effects against muscle atrophy and bone loss in a rat model of casting-induced muscle atrophy. It partially reversed gastrocnemius muscle wasting and helped preserve bone mineral density and microarchitecture, indicating its potential as an exercise-mimetic agent (Kang et al., 2019). However, in three placebo-controlled human trials (400 mg/day of UA for 8 weeks), supplementation did not confer additional gains in muscle mass or strength among resistance-trained young men consuming a high-protein diet (Lobo et al., 2021), nor did it improve metabolic syndrome parameters in postmenopausal women beyond combined moderate exercise, despite a modest increase in handgrip strength (Cione et al., 2021). Moreover, it failed to alter key inflammatory cytokines (e.g., TNF-α, IL6, and IL10) in healthy men undergoing resistance training (Lobo and Pimentel, 2022).

Docosahexaenoic acid (DHA) and eicosapentaenoic acid (EPA), typical ω-3 polyunsaturated fatty acids, also show potential therapeutic effects against muscle atrophy, particularly inflammation-induced muscle atrophy. In a study involving C2C12 myotubes, DHA and EPA were added before inducing inflammation with lipopolysaccharide (LPS). The results revealed that DHA and EPA treatments significantly counteracted the reduction in myotube diameter and myofibrillar protein content caused by LPS. Furthermore, in mouse myoblasts, these fatty acids suppressed the upregulation of muscle-specific atrophy markers, such as MAFbx and MuRF1 (Yamaguchi et al., 2021).

#### 6.1.3 Hormonal modulation

Hormonal modulation treatment strategies, particularly through testosterone and selective androgen receptor modulators (SARMs), are also being actively researched. Myostatin inhibitors, testosterone, and SARMs have been recognized for their ability to promote lean mass and potentially combat sarcopenia.

Testosterone supplementation in middle-aged and older men produces modest but significant gains in muscle mass and strength, though its impact on functional performance seems to be less clear. Across randomized trials, testosterone increased LBM by an average of 2.5 kg and improved handgrip strength by about 1.6 kg, while lower-limb strength measures rose by roughly 91 N in leg press and 144 N in leg extension. However, pooled data from common physical performance tests did not show a significant benefit (Parahiba et al., 2020). Thus, androgen therapy can counteract key components of sarcopenia, particularly muscle quantity and force.

SARMs demonstrate clear benefits for physical performance and body composition in adults (mean age: 57 years) over treatment periods of roughly 2–6 months. They increase stair-climbing power and one-repetition-maximum leg press strength, improve Short Physical Performance Battery scores, boost LBM, and slightly reduce fat mass. These functional and compositional gains occur alongside a moderate incidence of mild to moderate adverse events and a very low rate of serious complications, indicating that SARMs are generally well tolerated (Wen et al., 2025). The side effects of SARMs resemble those reported with testosterone use, but the degree is much less pronounced (Fonseca et al., 2020).

However, the long-term efficacy and safety of these treatments require further investigation, especially in older populations (Hardee and Lynch, 2019; Sakuma et al., 2023; Zheng et al., 2024). Other hormones like dehydroepiandrosterone, tibolone, estrogen, growth hormone, and ghrelin are also being explored for their role in enhancing muscle strength and function, with some showing initial benefits (Zheng et al., 2024). Nonetheless, the clinical evidence supporting hormone supplementation in sarcopenia remains inconclusive, necessitating additional research to clarify its benefits and risks (Kwak and Kwon, 2019).

### 6.2 Targeting mitochondrial dysfunction

#### 6.2.1 Exercise

Endurance exercise places a high energetic demand on contracting skeletal muscle, stimulating both mitochondrial biogenesis and selective removal of damaged mitochondrial regions via mitophagy. Recent work based on mouse models has shown that a single bout of treadmill running activates Ampk/Ulk1 signaling during and immediately after exercise, driving the incorporation of dysfunctional mitochondria into autolysosomes about 6 hours into recovery. Contrary to many stress models, this exercise-induced mitophagy in healthy muscle does not rely on Pink1 stabilization at the outer mitochondrial membrane. This is most likely because acute exercise does not provoke sufficient membrane depolarization, unfolded-protein accumulation, or mtDNA damage to recruit Pink1. Instead, the Fundc1 gene has been shown to orchestrate the recruitment of Ulk1 and LC3 to the mitochondrial reticulum only in response to hypoxia. While Pink1/Parkin pathways may become important under more extreme or pathological conditions (e.g., exhaustive exercise, aging, insulin resistance), maintaining mitochondrial quality in non-diseased muscle appears to depend on Ampk/Ulk1-driven mitophagy through a Pink1-independent mechanism (Drake et al., 2019).

Resistance training (RT) and mixed training (MT, combining RT with aerobic/balance exercises) are the most effective interventions for improving muscle strength (e.g., knee extension strength, grip strength) and physical performance (e.g., gait speed, timed up-and-go test) in older adults with sarcopenia, as demonstrated by multiple meta-analyses (Bao et al., 2020; Lu et al., 2021; Shen et al., 2023). While RT alone shows robust effects on strength and function, MT provides additional benefits for quality of life and mobility when combined with nutritional support (Shen et al., 2023). Whole-body vibration training (WBVT) exhibits limited efficacy, whereas mind-body exercises (e.g., Tai Chi) improve balance and functional mobility but not muscle mass (Lu et al., 2021; Wang et al., 2022). Optimal exercise protocols involve 3–5 sessions per week (30–80 min each) for ≥12 weeks, with RT/MT prioritized for muscle function and mind-body exercises for frailty-related balance issues (Bao et al., 2020; Wang et al., 2022). However, no exercise modality significantly increases muscle mass, which underlies the importance of combined approaches (e.g., exercise + nutrition) in sarcopenia management (Bao et al., 2020; Lu et al., 2021; Wang et al., 2022; Shen et al., 2023).

AMPK can be strongly activated in skeletal muscle by repeated muscle contractions and exercise (Thomson, 2018). Aging-related declines in AMPK and PGC-1α signaling may impair mitochondrial biogenesis, mitophagy, and dynamics, contributing significantly to skeletal muscle atrophy. Regular aerobic exercise effectively enhances the AMPK/PGC-1α signaling pathway, thereby improving mitochondrial function, promoting mitochondrial biogenesis and mitophagy, and attenuating sarcopenia, highlighting its therapeutic potential for combating age-related muscle degeneration (Liang et al., 2021).

#### 6.2.2 Nutritional and pharmacological interventions

Nutritional and pharmacological interventions have been explored for their potential to activate mitophagy and improve muscle function in the aging population. Several clinical studies (Andreux et al., 2019; Liu et al., 2022; Singh et al., 2022) have shown that daily oral administration of urolithin A at doses of 500–1,000 mg stimulates mitophagy and enhances mitochondrial metabolism in skeletal muscle, leading to greater resistance to fatigue, increased muscle strength, and markedly improved exercise performance.

Curcumin, another natural compound, has been shown to protect against muscle atrophy, reduce oxidative stress, and maintain mitochondrial function, making it a promising candidate for sarcopenia management (Mirzaei and Nazemi, 2022; Yusuf et al., 2022; Saud Gany et al., 2023). Of all natural supplements, curcumin shows enormous promise. However, its optimal dosage, delivery methods, and safety profile still require further investigation (Saud Gany et al., 2023).

Astragaloside IV (AS-IV), a bioactive compound from *Astragalus mongholicus* (Huangqi), has shown significant therapeutic potential for treating muscle diseases by targeting mitochondrial dysfunction. Through the PINK1/Parkin pathway, AS-IV reduces oxidative stress and regulates mitophagy, which is beneficial in maintaining mitochondrial integrity and function. In rat models, AS-IV can significantly reduce harmful ROS and malondialdehyde (MDA) levels, enhance superoxide clearance via Sod1, increase intracellular ATP concentrations, and thereby alleviate cellular oxidative stress (Li et al., 2023). Additionally, AS-IV modulates the expression of key mitochondrial proteins, ultimately restoring normal mitochondrial function and protecting muscle cells from damage.

An *in vivo* study Zhao et al. (2024) explored the potential of D-pinitol (DP) in treating diabetic sarcopenia by regulating mitophagy, with a focus on milk fat globule-EGF factor 8 protein (Mfge8). In diabetic mouse models, DP administration improved muscle function, reduced muscle atrophy, and enhanced mitophagy. DP decreased the levels of Mfge8, which is typically upregulated in sarcopenia, and promoted mitophagy by upregulating key proteins like Pink1 and Parkin, while reducing p62. Additionally, experiments indicated that DP and Mfge8 siRNA could stimulate mitophagy and alleviate symptoms of sarcopenia.

In a mouse model of denervation-induced muscle atrophy, celecoxib attenuated muscle wasting by reducing inflammation, oxidative stress, and protein degradation, while enhancing blood flow and promoting muscle regeneration (Zhang L. et al., 2022). Its protective effects are mediated by inhibiting COX2, suppressing ROS, and modulating key pathways involved in muscle proteolysis and mitophagy.

#### 6.2.3 Gene therapy

In aged mouse tibialis anterior muscle, overexpression of PGC-1α via *in vivo* DNA transfection markedly attenuated the age-related surge in mitophagy and fission markers, thus reducing PINK1 and Parkin levels by over 50%, lowering mitochondrial ubiquitination, and blunting increases in LC3, p62, Beclin-1 (Becn1, Atg6), Rheb, Mfn2, Fis1, and Drp1. This intervention also restored mitochondrial function, boosting citrate synthase activity, cytochrome c oxidase subunit IV (Cox4) levels, and antioxidant enzyme activities, while decreasing lipid peroxidation and inner membrane damage in old mice (Yeo et al., 2019). Despite these improvements in mitochondrial quality control and bioenergetics, PGC-1α overexpression did not reverse the muscle fiber atrophy associated with aging.

## 7 Conclusions and future prospects

This review outlines the multifaceted mechanisms by which aging drives a gradual yet relentless decline in muscle health. Diagnostic criteria have evolved over the past decade, moving from an initial focus on muscle mass to prioritizing muscle strength and physical performance. Major guidelines, such as the EWGSOP1, EWGSOP2, AWGS 2014, AWGS 2019, IWGS, and FNIH, provide clear diagnostic thresholds applicable to different populations, reflecting ethnic and regional variability in muscle characteristics and healthcare infrastructure.

Prevalence studies highlight sarcopenia as a widespread issue among aging populations, with considerable variation due to differences in diagnostic criteria, regional factors, and population demographics. Prevalence rates generally increase significantly after the age of 80 years, with notable differences between genders and ethnic groups. Higher prevalence rates in non-Asian compared with Asian populations suggest lifestyle factors, dietary habits, and physical activity levels play protective roles against sarcopenia.

The complex pathophysiology underlying sarcopenia involves several interconnected biological mechanisms. Hormonal changes, notably declines in testosterone and estrogen, substantially impact muscle metabolism and satellite cell activity, promoting muscle loss. Additionally, the activation of myostatin, a negative regulator of muscle growth, exacerbates muscle atrophy in response to hormonal shifts.

Muscle loss in aging individuals partly results from an imbalance between muscle protein synthesis and degradation, with age-related anabolic resistance reducing the muscle’s responsiveness to nutritional and exercise stimuli. mTOR functions as an amino acid sensing signaling hub that drives muscle protein synthesis and growth, but with aging and inactivity, its phosphorylation signaling is impaired, blunting the anabolic response to protein ingestion. Proteolytic systems, like the UPS and ALP, become excessively active in aged muscle, further accelerating muscle atrophy.

Chronic inflammation also significantly contributes to sarcopenia by disrupting muscle metabolism and regeneration. Elevated pro-inflammatory cytokines such as TNF-α and IL6 interfere with muscle protein synthesis and increase muscle breakdown, creating a detrimental environment that reduces muscle strength and regeneration capacity. Cellular senescence, characterized by an accumulation of senescent cells and their inflammatory secretome, further exacerbates muscle deterioration and impaired regeneration.

Mitochondrial dysfunction is pivotal in sarcopenia development. Aging-associated declines in mitochondrial quality and biogenesis, increased oxidative stress, and impaired mitophagy collectively compromise muscle energy metabolism and integrity. Excessive ROS production damages muscle cells, initiating cascades of mitochondrial and cellular dysfunction, ultimately leading to muscle fiber loss and functional impairment.

Targeting mitochondrial dysfunction and aging pathways offers promising therapeutic strategies for managing sarcopenia. Even though preclinical and early clinical studies demonstrate encouraging results in reducing oxidative stress, increasing muscle mass, and improving physical function, robust evidence regarding optimal dosing, long-term safety, and synergistic therapeutic combinations remains essential.

Future research should focus on integrating precision medicine approaches to sarcopenia diagnosis and management, considering regional, demographic, and individual variability. Developing reliable biomarkers, such as circulating indicators of mitophagy, exerkine profiles, mtDNA mutations, and advanced imaging-based markers, will be essential for early detection and real-time monitoring of therapeutic effectiveness. Additionally, combining exercise interventions with targeted nutritional and pharmacological treatments may optimize outcomes. Overall, deepening our understanding of sarcopenia’s multifactorial nature and implementing individualized, comprehensive interventions will be key to mitigating this significant age-related condition.

Ultimately, effectively combating sarcopenia demands a holistic strategy that safeguards mitochondrial function, balances muscle anabolism and catabolism, and corrects age-related hormonal and inflammatory imbalances. By combining in-depth scientific understanding, rigorous clinical research, and personalized therapeutic strategies, it is possible to slow the progression of muscle loss and substantially improve muscle strength, functional capacity, and independence among older adults.
